# AMH type II receptor and AMH gene polymorphisms are not associated with ovarian reserve, response, or outcomes in ovarian stimulation

**DOI:** 10.1007/s10815-016-0711-7

**Published:** 2016-05-03

**Authors:** Christian Cerra, William G. Newman, Dalia Tohlob, Helen Byers, Gregory Horne, Stephen A. Roberts, Lamiya Mohiyiddeen

**Affiliations:** 1 Department of Reproductive Medicine, St. Mary‘s Hospital, Central Manchester University Hospitals NHS Foundation Trust, Manchester, M13 9WL UK; 2 Manchester Centre for Genomic Medicine, Manchester Academic Health Sciences Centre (MAHSC), University of Manchester, Manchester, M13 9WL UK; 3 Manchester Centre for Genomic Medicine, Central Manchester University Hospitals NHS Foundation Trust, Manchester, M13 9WL UK; 4 Clinical pathology, Mansoura University Faculty of Medicine, Mansoura, Egypt; 5 Clinical Embryology, Department of Reproductive Medicine, St. Mary’s Hospital, Manchester, UK; 6 Centre for Biostatistics, Institute of Population Health, Manchester Academic Health Sciences Centre (MAHSC), University of Manchester, Manchester, UK

**Keywords:** Anti-Mullerian hormone, Antral follicle count, Follicle-stimulating hormone, Pharmacogenetics, Ovarian response, Ovarian hyperstimulation syndrome

## Abstract

**Purpose:**

Genetic variation may influence women’s response to ovarian stimulation therapy. The purpose of this study was to investigate any effects of genetic variants in the anti-Müllerian hormone (AMH) and AMH type II receptor genes on ovarian response/treatment outcomes and on current markers of ovarian reserve in individuals undergoing in vitro fertilisation (IVF) treatment.

**Methods:**

In this prospective observational study, we genotyped the *AMH* c.146G>T, p.(Ile49Ser) and *AMHR2* -482A>G variants in 603 unrelated women undergoing their first cycle of controlled ovarian stimulation for IVF and ICSI (intracytoplasmic sperm injection) using gonadotrophins at a tertiary referral centre for reproductive medicine. Pelvic ultrasound and blood hormone levels were taken on days 2–3 of the cycle. Genotypes were determined using TaqMan allelic discrimination assay. Regression analysis was performed to assess the relationship between the genotypes and the ovarian reserve markers (FSH, AMH, antral follicle count) and the early outcomes of response (number of oocytes retrieved and gonadotropin dose) as well as the treatment outcome (live birth).

**Results:**

There were no significant associations between the variants *AMH* c.146G>T and *AMHR2* -482A>G with ovarian response in terms of number of oocytes retrieved (*p* = 0.08 and *p* = 0.64, respectively), live births (*p* = 0.28 and *p* = 0.52) and/or markers of ovarian reserve.

**Conclusions:**

Genotyping of the *AMH* c.146G>T and *AMHR2* -482A>G polymorphisms does not provide additional useful information as a predictor of ovarian reserve or ovarian response and treatment outcomes.

## Introduction

Ovarian response to stimulation with exogenous gonadotrophins during assisted reproductive techniques (ART) can range from an inadequate response, which may result in cycle cancellation, to a high response, which can put women at risk of ovarian hyperstimulation syndrome (OHSS).

Markers of ovarian reserve, such as basal serum FSH (follicle stimulating hormone) levels, estradiol, inhibin B, anti-Müllerian hormone (AMH) levels and antral follicle count (AFC), are currently used in clinical practice to predict response to stimulation [1]. Of these markers, AMH has the advantage of having relatively stable levels throughout the menstrual cycle [2, 3] and is more accurate in providing information on the expected ovarian response to controlled stimulation [3, 4]. However, it is not highly predictive of extremes of responses, and its value has been questioned [5].

AMH is a member of the transforming growth factor beta superfamily and is produced by the granulosa cells of pre-antral and small antral follicles in the ovary. It is highly expressed in the developing follicles, but its expression diminishes at the stage for selection of the dominant follicle by FSH [6]. It is used in clinical practice as a biomarker of ovarian reserve [6]. AMH is expressed in granulosa cells and may regulate FSH sensitivity in the ovary [7]. Therefore, it has been proposed to have a role in influencing ovarian response to stimulation [6]. It exerts its biological effects through the receptor AMHR2, which is present on granulosa and theca cells.

Considering the important role of the AMH signalling pathway in regulating FSH sensitivity in the ovary and follicular recruitment and selection, it is appropriate to consider that variation in the genes encoding key proteins in the pathway may influence ovarian response. Two polymorphisms, in *AMH* c.146G>T, p.Ile49Ser (rs10407022) and AMH type II receptor (*AMHR2*) -482A>G (rs2002555) genes, have been genotyped in a number of studies. These two polymorphisms have been associated with increased follicular phase oestradiol levels [7], menopausal age related to parity [7–9], age at natural menopause [10], and severity of the polycystic ovary syndrome (PCOS), in terms of follicle number and androgen levels [11], and unexplained infertility [12]. Three previous studies have considered these polymorphisms in relation to their direct effects during ovarian stimulation in ART treatment. A study of 191 women did not find a statistically significant association between these polymorphisms and response to ovarian stimulation [13]. In the second study of 151 women by Karagiorga et al., significant differences between certain subgroups following controlled ovarian stimulation were found amongst women wild type for the *AMH* polymorphism, in those with more than two previous IVF attempts, basal serum FSH levels were lower (*p* = 0.012), and in those with lower peak serum E2 levels, fertilisation rates were higher (*p* = 0.037) [14]. In addition, women wild type for the *AMHR2* polymorphism with more than two previous IVF attempts had a higher number of follicles, and women with higher peak serum E2 levels required a lower gonadotropin dose [14]. The third study of 186 infertile women by Peluso et al. showed an association between *AMHR2* polymorphisms and FSH, estradiol and AMH levels, whilst *AMH* polymorphisms were associated with the number of embryos produced [15].

Given the differences in the previous reports, we undertook a study in a previously collected and characterised cohort of patients [15]. We genotyped *AMH* c.146G>T, p.(Ile49Ser) and *AMHR2* -482A>G in 603 unrelated women who underwent ovarian stimulation using gonadotrophins and correlated genotypes with ovarian reserve markers and treatment outcomes.

## Materials and methods

### Subjects and assays

A total of 603 women undergoing assessment prior to their first IVF treatment cycle were recruited to the study. Inclusion criteria for the study were as follows: [1] presence of both ovaries [2], <40 years of age [3], no use of hormone therapy in the 6 months preceding the recruitment [4], no previous ovarian surgery or pelvic radiation therapy [5], commencing first cycle IVF treatment. In addition, the women had a body mass index (BMI) >19 and <30 kg/m^2^ to satisfy eligibility criteria for government-funded IVF treatment in Greater Manchester, UK [16]. The protocol was approved by the South Manchester Research Ethics Committee (REC ref no. 08/81003/212). Written informed consent was obtained from all participants.

### Baseline evaluation

On days 2 to 3 of a spontaneous menstrual cycle or after a withdrawal bleed in anovulatory women, blood samples for measurement of FSH and AMH levels were obtained by venepuncture.

The FSH concentration was measured using specific immunoassay kits (Cobas; Roche Diagnostics). The intra-assay and inter-assay coefficients of variations were 2.6 and 2.8 %, respectively.

AMH levels were measured and determined by first generation ELISA provided by DSL (Oxford Bio Innovation). The functional sensitivity of the assay was 0.05 ng/mL. Intra- and inter-assay coefficients of variation were <5 and <5.5 %, respectively. Conversion factor to ng/mL = pmol/L ÷ 7.143.

AMH was used in treatment individualisation and is presented in the three triage bands (15.6 pmol/L, 15.6–28.6 pmol/L, >28.6 pmol/L) used for clinical decision-making and allocation to a treatment protocol at that time.

On days 2–3 of a spontaneous menstrual cycle within 3 months of commencing ovarian stimulation, a transvaginal ultrasound scan was performed to assess the total number of antral follicles measuring 2–5 mm in diameter and to confirm normal anatomy of the pelvic organs. Intra-analysis coefficient of variation for follicular diameter measurements was <5 %, and the lower limit of detection was 0.5 mm.

### IVF treatment protocol

Patients underwent either standard long downregulated cycles using gonadotrophin-releasing hormone (GnRH) analogues or short cycles using GnRH antagonists. The GnRH analogue (buserelin acetate; Aventis Pharma Ltd) was administered at the dose of 0.5 mg subcutaneously starting from the midluteal phase of the preceding menstrual cycle. Ovarian stimulation was effected with exogenous recombinant FSH (Puregon; Organon Laboratories Ltd) or highly purified FSH (Menopur; Ferring Pharmaceuticals). The GnRH analogue was reduced to 0.25 mg from the first day of gonadotrophin stimulation.

Patients on the antagonist cycle had gonadotrophin stimulation initiated on day 2 of the cycle, continuing up to and including the day of human chorionic gonadotrophin administration; the GnRH antagonist (Orgalutran; Organon Laboratories) at a daily dose of 0.25 mg was initiated using a fixed day-6 protocol.

Transvaginal ultrasounds were arranged on days 8 and 10, then daily or on alternate days thereafter as required. Final oocyte maturity was induced with 5000 IU of hCG (Pregnyl; Organon Laboratories) in the presence of more than three follicles of R17 mm. Oocyte retrieval was performed 34 to 36 hours after hCG administration, and a maximum of two embryos was transferred 3 days later. Vaginal progesterone pessaries (Cyclogest, 400 mg twice daily; Alpharma) were used to support the luteal phase. We adopted standard stimulation protocols across the groups.

Poor response was defined as the collection of less than four oocytes at retrieval or cancellation of the cycle when fewer than three mature follicles had developed. A normal response was considered to be retrieval of 4 to 20 oocytes, and over-response defined as >20 oocytes retrieved. Clinical pregnancy was defined as the presence of a gestation sac containing a foetal pole and foetal heart activity seen on transvaginal scan from 6 weeks of gestation.

### Genotyping

DNA was extracted from blood samples using the Chemagen Automated DNA Separation System. Genotypes for *AMH* c.146G>T, p.(Ile49Ser) (rs10407022) and *AMHR2* -482A>G (rs2002555) were determined using the TaqMan SNP pre-designed genotyping assay (assays ID C_25599842_10 and C_1673084_10; Applied Biosystems).

### Statistical analysis

Statistical analysis was performed using Rv2.15 (R Development CoreTeam, 2013). Kruskall-Wallis and Fisher’s exact tests were used to compare characteristics across different genotypes.

Linear regression analysis was performed to assess the effect of the *AMH* c.146G>T and *AMHR2* -482A>G genotypes, represented as an allele number, on the log of ovarian reserve markers (s-FSH, AFC and s-AMH), with adjustment for age (as a 4 degree of freedom cubic spline) and BMI (as a linear effect) and on the primary outcomes of response (gonadotrophin dose and log of number of oocytes retrieved) with adjustment for age, BMI and treatment (s-AMH treatment band, stimulation type and their interaction). s-AMH was used to select the treatment, hence its inclusion as a treatment parameter here. In addition, we also considered live birth and a dichotomous measure of over-response of >20 oocytes retrieved (used in a previous similar study [15]), which we analysed using analogous logistic regression models. There were no cycles cancelled for over-response, and any cycles cancelled for under-response were recorded as having yielded no oocytes.

A *P* value of <0.05 was considered statistically significant.

This study was performed using a previously collected and characterised sample; therefore, there was no formal sample size computation used to design the study. With genotypes containing the minor alleles forming approximately one-third of the sample, we have >80 % power to detect small effect sizes of 0.25 (in terms of the standard deviation between patients) or odds ratios of around 0.6 for a live birth rate of 30 %.

## Results

Complete clinical data were available for 560 women: 19 were excluded as they never proceeded to ovarian stimulation, and no blood sample was obtained from 24 individuals. Genotyping of *AMH* c.146G>T, p.(Ile49Ser) and *AMHR2* -482A>G variants was unsuccessful in 19 and 12 women, respectively.

Tables 1 and 2 show the characteristics/demographics for the women included in the analysis genotyped for *AMH* c.146G>T, p.(Ile49Ser) and *AMHR2* -482A>G, respectively. There were no statistically significant differences in genotype frequencies with respect to BMI, duration of infertility or ovarian stimulation type. The one borderline significant association with AMHR2 and age (*P* = 0.041) would not be considered significant after allowing for the number of characteristics tested. Different allele frequencies in different ethnic groups were noted for *AMH* c.146G>T; therefore, we performed a separate analysis for the largest ethnic group (“White”), not presented here, which gave results consistent with the analysis for the whole group presented below.

**Table 1:** Clinical characteristics of women undergoing IVF treatment by *AMH* genotype

	*AMH* c.146G>T, p.(Ile49Ser)			*n* = 541
Characteristic	G/G *n* = 24 (4 %)	G/T *n* = 176 (33 %)	T/T *n* = 341 (63 %)	*P*
Age (years)	35.3 (25.7–38.7)	33.2 (25.7–37.7)	33.3 (26.6–38.3)	0.06
BMI	23.1 (20–26.9)	24.1 (19.8–29.1)	23.7 (20.2–29.6)	0.26
Duration of infertility (years)	4 (2–7)	3 (2–7)	3 (2–6)	0.55
Ethnicity				
White South Asian Black Other	12 (50 %) 7 (29 %) 3 (12 %) 2 (8 %)	142 (81 %) 27 (15 %) 4 (2 %) 3 (2 %)	306 (90 %) 25 (7 %) 5 (1 %) 5 (1 %)	<0.001
Primary infertility cause				
Male factor Tubal factor Endometriosis Ovarian Unexplained	7 (29 %) 5 (21 %) 0 5 (21 %) 7 (29 %)	53 (30 %) 44 (25 %) 7 (4 %) 21 (12 %) 50 (29 %)	108 (32 %) 87 (26 %) 18 (5 %) 28 (8 %) 99 (29 %)	0.93 0.95 0.66 0.07 0.99
Ovarian stimulation type				
Agonist Antagonist	4 (17 %) 20 (83 %)	69 (39 %) 107 (61 %)	111 (33 %) 229 (67 %)	0.061

Note: values are in median (10th–90th percentile) or numbers (percentages of women with genotype)

*P* is from Fisher’s exact test or Kruskall-Wallis test

**Table 2:** Clinical characteristics of women undergoing IVF treatment by *AMHR2* genotype

	*AMHR2* -482A>G			*n* = 548
Characteristic	A/A *n* = 379 (69 %)	A/G *n* = 152 (28 %)	G/G *n* = 17 (3 %)	*P*
Age (years)	33.1 (26.1–38.1)	33.9 (27.4–38.0)	30.8 (25.9–35.9)	0.041
BMI	23.8 (20.1–29.2)	23.9 (20.0–29.5)	23.8 (20.5–29.2)	0.99
Duration of infertility (years)	3 (2–7)	4 (2–6)	2 (2–6)	0.60
Ethnicity				
White South Asian Black Other	322 (85 %) 40 (11 %) 9 (2 %) 8 (2 %)	129 (85 %) 18 (12 %) 3 (2 %) 2 (1 %)	15 (88 %) 2 (12 %) 0 0	0.98
Primary infertility cause				
Male factor Tubal factor Endometriosis Ovarian Unexplained	113 (30 %) 95 (25 %) 20 (5 %) 42 (11 %) 108 (29 %)	49 (32 %) 40 (26 %) 5 (3 %) 12 (8 %) 45 (30 %)	8 (47 %) 1 (6 %) 1 (6 %) 1 (6 %) 6 (35 %)	0.29 0.17 0.49 0.61 0.78
Ovarian stimulation type				
Agonist Antagonist	128 (34 %) 251 (66 %)	53 (35 %) 99 (65 %)	11 (69 %) 8 (47 %)	0.97

Note: values are in median (10th–90th percentile) or numbers (percentages of women with genotype)

*P* is from Fisher’s exact test or Kruskall-Wallis test

There were no statistically significant associations between the measures of ovarian reserve (basal s-FSH, AFC and s-AMH) and the *AMH* c.146G>T or *AMHR2* -482A>G genotype groups, after adjusting for age, ethnicity and BMI (Tables 3 and 4). There were also no significant associations between the genotypes and outcomes of ovarian response (in terms of number of oocytes and embryos retrieved, gonadotrophin dose and live birth rate) after adjustment for age, BMI and treatment.

**Table 3:** Ovarian response and outcomes and markers of ovarian reserve by AMH genotype

	*AMH* c.146G>T, p.(Ile49Ser)			Adjusted^a,b^	
	G/G (*n* = 24)	G/T (*n* = 176)	T/T (*n* = 341)	Change per allele (95 % CI)^b^	*P*
Ovarian response outcomes					
No. of oocytes retrieved	11	9	9	−0.09	0.08
	(3–18)	(3–20)	(3–18)	(−0.18–0.01)	
No. of embryos	5	4	4	0.01	0.87
	(0–12)	(0–10)	(0–9)	(−0.10–0.12)	
Gonadotrophin dose (IU)	2100	2700	2700	74	0.30
	(1200–3435)	(1500–3725)	(1400–3750)	(−66–215)	
Live birth	7/24	51/176	92/341	0.83	0.28
	(29 %)	(29 %)	(27 %)	(0.58–1.17)	
Under-response (<4 oocytes)	3/24	23/176	46/341	1.09	0.70
	(12 %)	(13 %)	(13 %)	(0.69–1.74)	
Over-response (>20 oocytes)	1/24	16/176	18/341	0.77	0.42
	(4 %)	(9 %)	(5 %)	(0.4–1.47)	
Ovarian reserve					
	7.0	7.0	6.6	−0.03	0.35
s-FSH (IU/L)	(4.5–10.8)	(4.7–10.4)	(4.6–9.8)	(−0.08–0.03)	
AFC	15	13	13	0.01	0.75
(2–5 mm)	(6.4–31)	(8–21)	(8–24)	(−0.06–0.08)	
s-AMH (pmol/L)	12.9	14.5	15.7	0.06	0.32
	(6.0–49.5)	(5.5–37.1)	(5.1–42.4)	(−0.06–0.17)	

Note: Outcome values are median (10th–90th percentile), effect sizes shown with 95 % CI

^a^Adjusting for age as cubic spline and BMI as linear effect, ethnicity as four groups, with oocyte and embryo number, s-FSH, AFC and s-AMH being log-transformed. Effect size is change in (log) value per allele for the continuous variables or odds ratios for live birth and under/over-response

^b^Response outcomes additionally adjusted for treatment as AMH treatment band, stimulation type and their interaction

**Table 4:** Ovarian response and outcomes, and markers of ovarian reserve by AMHr2 genotype

	*AMHR2* -482A>G			Adjusted^a,b^	
	A/A (*n* = 379)	A/G (*n* = 152)	G/G (*n* = 17)	Change per allele (95 % CI)^b^	*P*
Ovarian response outcomes					
No. of oocytes retrieved	9	8	10	0.02	0.64
	(3–18)	(3–18)	(3–16)	(−0.08–0.12)	
No. of embryos	4	4	4	0.09	0.20
	(0–9)	(1–10)	(0–9)	(−0.02–0.21)	
Gonadotrophin dose (IU)	2700	2475	3000	36	0.63
	(1350–3750)	(1428–3600)	(1710–3300)	(−110–183)	
Live birth	103/379	43/152	6/17	1.12	0.52
	(27 %)	(28 %)	(35 %)	(0.79–1.60)	
Under-response (<4 oocytes)	55/379	17/152	2/17	0.82	0.44
	(15 %)	(11 %)	(12 %)	(0.49–1.37)	
Over-response (>20 oocytes)	25/379	10/152	0/17	0.83	0.59
	(7 %)	(7 %)	(0 %)	(0.41–1.65)	
Ovarian reserve					
s-FSH (IU/L)	6.8	6.7	6.2	−0.02	0.50
	(4.6–10.1)	(4.6–10.0)	(4.9–9.2)	(−0.07–0.04)	
AFC (2–5 mm)	13	13	14	−0.01	0.69
	(8–23)	(8–20)	(10–31)	(−0.08–0.05)	
s-AMH (pmol/L)	15.3	14.7	24.7	0.04	0.49
	(5.3–42.6)	(5.2–36.0)	(6.0–63.2)	(−0.08–0.16)	

Note: Outcome values are median (10th–90th percentile), effect sizes shown with 95 % CI

*AMH* anti-Mullerian hormone, *BMI* body mass index, *FSH* follicle-stimulating hormone

^a^Adjusting for age as cubic spline and BMI as linear effect, ethnicity as four groups, with oocyte and embryo number, s-FSH, AFC and s-AMH being log-transformed. Effect size is change in (log) value per allele for the continuous variables or odds ratios for live birth and under/over-response

^b^Response outcomes additionally adjusted for treatment as AMH treatment band, stimulation type and their interaction

A dichotomous measure of response (>20 oocytes for high response) was similarly analysed using equivalent logistic regression models. We did not find a statistically significant association between the *AMH* c.146G>T and *AMHR2* -482A>G variants and high response to ovarian stimulation adjusted for age, ethnicity and BMI (per allele odds ratio (OR) = 0.77, 95 % confidence interval (CI) = 0.4–1.47 for *AMH* c.146G>T and OR = 0.83, CI = 0.41–1.65 for *AMHR2* -482A>G, respectively).

## Discussion

Controlled ovarian hyperstimulation in assisted reproduction involves the use of exogenous gonadotrophins, which can result in an excessive response including OHSS, or inadequate response leading to cycle cancellation. This significant variability in response has been the focus of many pharmacogenetic studies investigating single nucleotide polymorphisms (SNPs) as markers of ovarian reserve and predictors of ovarian response in order to optimise and individualise treatments.

Serum AMH levels are commonly used in current clinical practice as markers of ovarian reserve and to predict the response to controlled ovarian stimulation.

This large study investigated the association between the *AMH* c.146G>T, p.(Ile49Ser) and *AMHR2* -482A>G polymorphisms and ovarian response in women undergoing IVF treatment. In addition, we included the most important clinical outcome, live birth rate, as an outcome measure. We did not identify any statistically significant differences (*P* < 0.05) in ovarian reserve markers and in treatment outcomes, including the number of oocytes retrieved and live birth rates between women with different *AMH* or *AMHR2* genotypes, after adjusting our results for age, BMI, stimulation protocol and s-AMH band used to allocate treatment.

The study by Hanevik et al. similarly did not demonstrate any association between the *AMH* and *AMHR2* polymorphisms and high or low response to ovarian stimulation [13]. Further, our study considered additional outcomes and markers of ovarian reserve.

Peluso et al. found in their study of 186 women that *AMH* polymorphisms were associated with the number of embryos produced and that *AMHR2* polymorphisms were associated with the ovarian reserve markers (AMH, FSH levels and antral follicle count). However, they did not specify if any adjustments were made in their logistic regression model and so a direct comparison of the results is difficult.

Differences in results between association studies may be due to different allelic frequencies between ethnic groups. We did note differences in the allele frequencies between the ethnic groups recruited to our study and so performed a further analysis considering the dichotomous variables (over/under response) for the largest group (white British individuals) as per the previous study [13]. No significant associations were determined, indicating that it is unlikely that our combined analysis masked an association specific to a discrete ethnic group. Further, the allelic distributions we observed were similar to those found in Brazilian [Peluso et al.], Greek [Karagiorga et al.] and Norwegian [Hanevik et al.] cohorts.

Other factors that may explain the lack of consensus amongst the different studies that have investigated relationships between *AMH* and *AMHR2* genotypes and controlled ovarian stimulation include differences in ovarian stimulation protocols used, the design of the study and the outcome measures. In the study by Karagiorga et al., significant associations were revealed only after subgroup analyses were performed based on age, AMH levels, peak E2 serum levels and number of IVF cycles. In addition, the outcome measure used in their study was different, as they compared number of follicles rather than number of oocytes retrieved used for our study.

Although the *AMH* and *AMHR2* variants were not associated with ovarian response in this study, Boudjenah et al. found that it was only the combination of *AMH* and *FSHR* variants which correlated with the number of mature oocytes produced during controlled ovarian stimulation with recombinant FSH [17].

In conclusion, when considering the development of integrative clinical algorithms for individual FSH doses, our results indicate that genotyping *AMH* c.146G>T and *AMHR2* -482A>G variants does not provide useful information as a predictor of response to ovarian stimulation and treatment outcomes.
